# Constitutively active form of natriuretic peptide receptor 2 ameliorates experimental pulmonary arterial hypertension

**DOI:** 10.1038/mtm.2016.44

**Published:** 2016-07-06

**Authors:** Nobutoshi Nawa, Hidekazu Ishida, Shinichi Katsuragi, Hiroki Baden, Kunihiko Takahashi, Ryota Higeno, Fumiko Torigoe, Seiko Mihara, Jun Narita, Kohji Miura, Kazufumi Nakamura, Shigetoyo Kogaki, Keiichi Ozono

**Affiliations:** 1 Department of Pediatrics, Graduate School of Medicine, Osaka University, Osaka, Japan; 2 Department of Cardiovascular Medicine, Graduate School of Medicine, Dentistry, and Pharmaceutical Sciences, Okayama University, Okayama, Japan

## Abstract

We recently found a constitutively active mutant of natriuretic peptide receptor 2 (caNPR2; V883M), which synthesizes larger amounts of cyclic guanosine monophosphate (cGMP) intracellularly without any ligand stimulation than existing drugs. The aim of this study was to investigate the therapeutic effects of gene transduction using ca*NPR2* for pulmonary arterial hypertension (PAH). *In vitro* gene transduction into human pulmonary arterial smooth muscle cells using Sendai virus (SeV) vectors carrying ca*NPR2* induced 10,000-fold increases in the synthesis of cGMP without ligand stimulation, and the proliferation of ca*NPR2*-expressing cells was significantly attenuated. The PAH model rats generated by hypoxia and the administration of SU5416 were then treated with SeV vectors through a direct injection into the left pulmonary artery. Right ventricular systolic pressure was significantly decreased 2 weeks after the treatment, while systemic blood pressure remained unchanged. Histological analyses revealed that the medial wall thickness and occlusion rate of pulmonary arterioles were significantly improved in ca*NPR2*-treated lungs. Neither the systemic integration of virus vectors nor side effects were observed. The massive stimulation of cGMP synthesis by gene therapy with ca*NPR2* was safe and effective in a PAH rat model and, thus, has potential as a novel therapy for patients with severe progressive PAH.

## Introduction

Pulmonary arterial hypertension (PAH) is a devastating disease with a 5-year survival rate of 61.2% for newly diagnosed WHO Group 1 PAH patients.^1^ Both the constriction of pulmonary vascular smooth muscles, and the proliferative and antiapoptotic characteristics of smooth muscle cells play an important role in the pathogenesis of PAH.^2,3^ In PAH patients, narrowed and obstructed small pulmonary arteries increase pulmonary vascular resistance, leading to right heart failure.^2^ At present, multiple drug therapies are clinically available. Among them, elevations in intracellular cyclic guanosine monophosphate (cGMP) concentrations have been suggested to effectively suppress proliferation and induce apoptosis in pulmonary arterial smooth muscle cells.^4,5^ Several strategies are used to increase intracellular cGMP concentrations: (i) inhibiting phosphodiesterase type 5 (PDE5) activities; (ii) activating soluble guanylyl cyclase (sGC); and (iii) activating particulate guanylyl cyclase. Several types of PDE5 inhibitors and a sGC stimulator are now clinically available. However, some patients are resistant to these medications and require lung transplantation. Therefore, a novel therapeutic strategy for severe PAH is strongly needed.

C-type natriuretic peptide (CNP) is a member of the natriuretic peptide family^6^ and is expressed in chondrocytes and vascular endothelial cells.^7,8^ Its receptor is natriuretic peptide receptor 2 (NPR2), which is expressed in chondrocytes and vascular smooth muscle cells.^8^ NPR2 is a transmembrane receptor that functions as a particulate guanylyl cyclase and increases cGMP concentrations upon ligand binding. The CNP/NPR2 signaling pathway has recently been regarded as a potent therapeutic target for PAH. A previous study showed that CNP was effective in a PAH rat model,^9^ while another reported that it was not,^10^ which may have been due to the down-regulation of NPR2 induced by the long-term infusion of CNP. We more recently identified a novel constitutively active mutant of NPR2 (caNPR2; Val883Met) in a family case showing overgrowth and bone anomalies.^11^ This caNPR2 has the ability to increase intracellular cGMP levels by 10,000-fold over those of the normal static state when transduced into a human cell line. This elevation in cGMP level was markedly higher than that achieved by a PDE5 inhibitor (several fold).^4,5^

Virus-based gene therapy has recently been revived the following several successful clinical trials for various diseases.^12–14^ Among the various types of virus vectors available, Sendai virus (SeV) vectors are very promising because of their low toxicity and high efficiency in gene transduction assessed by the clinical trials.^14,15^ We herein constructed a SeV vector carrying ca*NPR2* and aimed to investigate the therapeutic effects and safety of SeV vector–mediated gene therapy with ca*NPR2* for PAH using a Sugen PAH rat model and patient-derived pulmonary arterial smooth muscle cells.

## Results

### The ca*NPR2* synthesizes large amounts of cGMP and suppresses the proliferation of pulmonary arterial smooth muscle cells *in vitro*

**Figure 1:**
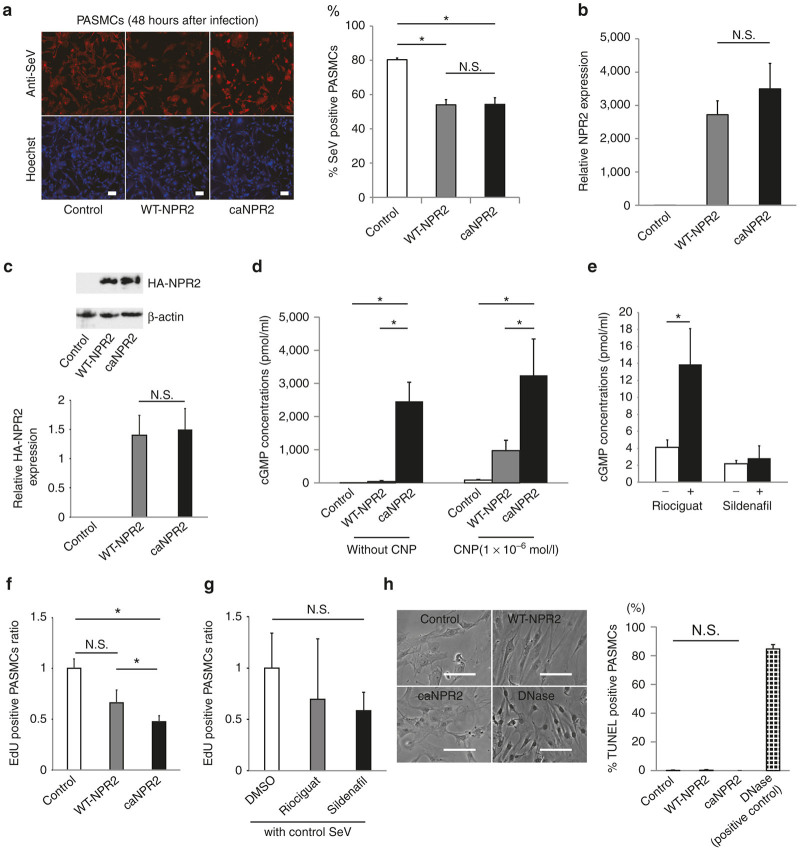
Transduction of the constitutively active mutant of natriuretic peptide receptor 2 (caNPR2) by Sendai virus (SeV) vectors in human pulmonary arterial smooth muscle cells (PASMCs). (a) Immunocytochemical analyses show that SeV vectors carrying Azami-Green control, wild-type *NPR2* (WT-*NPR2*), and ca*NPR2* efficiently infect PASMCs. The infectious ability of the Azami-Green-expressing control SeV vector (purchased as ready to use) is higher than that of the WT-*NPR2*- or ca*NPR2*-expressing vectors, with no significant difference between WT-*NPR2*- and ca*NPR2*-expressing SeV vectors (*n* = 3). Bar = 100 µm. *P < 0.05. N.S., not significant. (b) A quantitative PCR analysis shows no significant difference in *NPR2* expression levels between WT-*NPR2*- and ca*NPR2*-transduced PASMCs (*n* = 3). N.S., not significant. (c) Western blotting demonstrates that NPR2 expression levels were not different between WT-*NPR2*- and ca*NPR2*-transduced PASMCs, as detected by anti-HA tag antibody (*n* = 3). N.S., not significant. (d) The overexpression of ca*NPR2* induces the synthesis of larger amounts of cyclic guanosine monophosphate (cGMP) in PASMCs with and without the C-type natriuretic peptide (CNP) stimulation (*n* = 3). Note that ca*NPR2 *has the ability to produce large amounts of cGMP, regardless of the CNP stimulation. Even under the unphysiologically high concentration of CNP (10^−6^ mol/l), cGMP levels are higher in ca*NPR2*-expressing cells than in WT-*NPR2*-expressing cells. *P < 0.05. (e) The effects of riociguat (100 µmol/l) or sildenafil (5 µmol/l) on cGMP concentrations in PASMCs (*n* = 3 for riociguat; *n* = 8 for sildenafil). *P < 0.05. (f) The EdU incorporation assay reveals that ca*NPR2* has the ability to suppress the proliferation of PASMCs (*n* = 6). *P < 0.05. (g) The EdU incorporation assay reveals that riociguat (100 µmol/l) and sildenafil (5 µmol/l) did not have significant effects, although we found a small tendency toward attenuation of cell proliferation of PASMCs (*n* = 4). N.S., not significant. (h) The TUNEL assay demonstrates that ca*NPR2* does not induce apoptosis in PASMCs (*n* = 3). The left panels show representative phase contrast images. Bar = 500 µm. The right panel shows corresponding quantifications. N.S., not significant.

We initially conducted immunocytochemical, quantitative PCR (qPCR) and western blot analyses to confirm the gene transduction abilities of our SeV vector toward pulmonary arterial smooth muscle cells (PASMCs). Two days after the infection, immunostaining with an anti-SeV antibody clearly demonstrated that 50% of PASMCs were infected with SeV vectors carrying wild-type (WT)-*NPR2* and ca*NPR2*, whereas the infectious ability of the control SeV vector (Azami-Green-carrying SeV, which is purchased ready to use) was higher (Figure 1a). qPCR and western blot analyses showed successful transgene expression, and no significant differences were observed in the expression levels of NPR2 between WT-*NPR2*- and ca*NPR2*-transduced PASMCs (Figure 1b,c). In order to confirm the promotive effects of ca*NPR2* on cGMP synthesis, we measured intracellular cGMP concentrations in control (Azami-Green SeV), WT-*NPR2*-, and ca*NPR2*-transduced PASMCs. Even under the CNP-free condition, ca*NPR2*-expressing PASMCs synthesized large amounts of cGMP (control, 0.193 ± 0.115 pmol/ml; WT-*NPR2*, 38.3 ± 29.6 pmol/ml; ca*NPR2*, 2,460 ± 577 pmol/ml; *n* = 3; *P* < 0.01), which was consistent with our previous findings using the human cell line HEK293.^11^ We also evaluated cGMP concentrations in each transfected group under an unphysiologically strong CNP stimulation (10^–6^ mol/l). We found that ca*NPR2*-expressing cells still produced greater amounts of cGMP than WT-*NPR2*-transduced cells (Figure 1d). These results demonstrate that ca*NPR2* may force the synthesis of 10,000-fold higher amounts of cGMP under any ligand concentration condition. No obvious cell death was detected with the high intracellular cGMP concentrations induced by ca*NPR2*. In order to compare the therapeutic potential of ca*NPR2* with clinically available drugs, we measured intracellular cGMP concentrations in riociguat (a sGC stimulator)- and sildenafil (a PDE5 inhibitor)-treated PASMCs, which revealed that cGMP concentrations were 3.5- and 1.3-fold higher after two respective drugs compared with untreated PASMCs (Figure 1e). These results confirmed that the *in vitro* gene transduction of ca*NPR2* more strongly induced the synthesis of cGMP than the sGC stimulator and PDE5 inhibitor.

In order to examine the cellular physiology of PASMCs with high intracellular cGMP concentrations, cell proliferation was evaluated by EdU assays. The proliferative ability of PASMCs was significantly suppressed by ca*NPR2* transduction (Figure 1f). In contrast, riociguat or sildenafil did not have significant effects, although we found a small tendency toward attenuation of cell proliferation (Figure 1g). TUNEL assays and Annexin-V staining demonstrated that apoptosis of ca*NPR2*-expressing PASMCs was not affected (Figure 1h, Supplementary Figure S1). Because few apoptotic cells were detected under normal PASMC culture conditions, we induced apoptosis in cells using camptothecin, but still did not observe any significant differences between groups (data not shown).

### The direct pulmonary artery injection safely and successfully introduces SeV vectors into PASMCs *in vivo*

**Figure 2:**
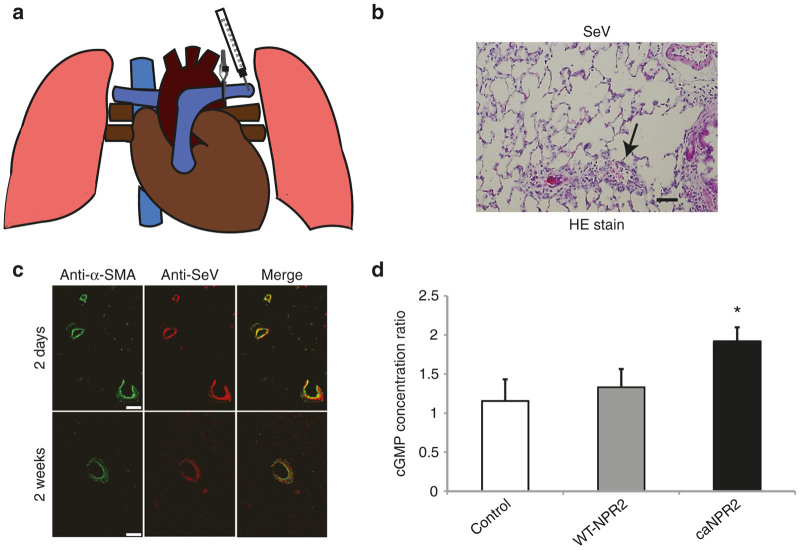
Intrapulmonary artery injection of Sendai virus (SeV) safely and efficiently transduces constitutively active mutant of natriuretic peptide receptor 2 (caNPR2) into pulmonary arterial smooth muscle cells *in vivo*. (a) A schematic image of an intravascular injection of SeV into the left pulmonary artery with clamping of the proximal site. (b) Hematoxylin and eosin staining shows that virus-induced tissue toxicity and inflammation are minimal (arrows) in SeV vector-induced lungs. Bar = 100 µm. (c) Immunohistochemical analyses show that SeV vectors are successfully transduced into the pulmonary vasculature and particularly into pulmonary arterial smooth muscle cells. SeV vectors are detected from 2 days until at least 2 weeks after the injection. Bar = 50 µm. (d) An intravascular injection of SeV carrying ca*NPR2* significantly increases cyclic guanosine monophosphate (cGMP) concentrations in the injected side (left) lung lysates (*n* = 3 for control; *n* = 3 for wild-type (WT)-*NPR2*; *n* = 5 for ca*NPR2*). Each cGMP concentration is normalized by the untreated side (right) lung lysate. *P < 0.05.

**Figure 3:**
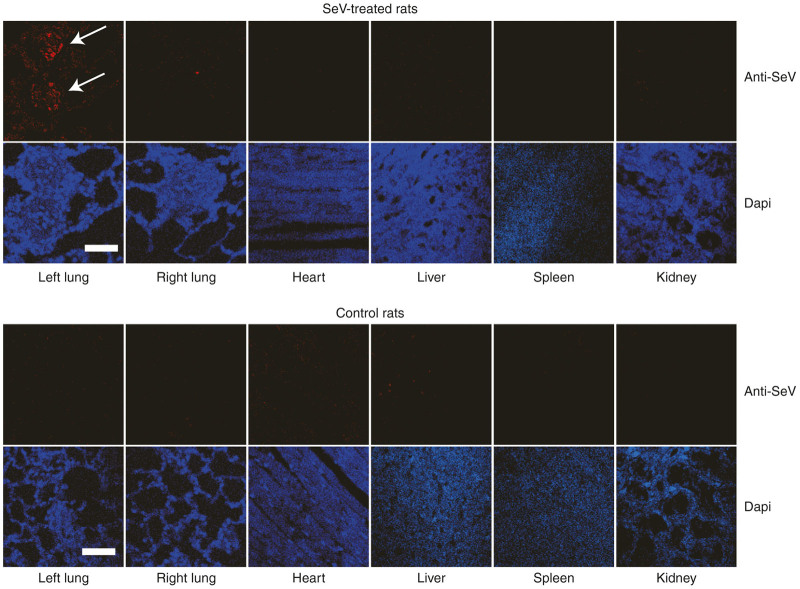
Immunohistocytochemistry for Sendai virus (SeV) vector infections in other organs. Two weeks after the injection of SeV vectors into the left pulmonary artery, no SeV antigen is found in the other organs, including the heart, liver, spleen, kidney, and right lung. These results suggest that the SeV vector injection into the left pulmonary artery with 2 minutes of clamping selectively transduces vectors into the vascular cells in the left lung (arrows). Bar = 50 µm.

We selected the direct injection of SeV vectors into the proximal pulmonary artery for the efficient introduction of ca*NPR2* into smooth muscle cells in small pulmonary arteries (mainly the resistant arteries). After left lateral thoracotomy, we clamped the proximal left pulmonary artery and injected the SeV vector solution into the distal side of the artery (Figure 2a). By interrupting blood flow with the small clamping device, SeV vectors effectively infected the pulmonary artery walls. Hematoxylin and eosin staining of lung tissues demonstrated that virus-induced tissue toxicity and inflammation were minimal in SeV vector-treated lungs via an intrapulmonary artery injection (Figure 2b). Immunohistochemical analyses using anti-SeV and anti-α-smooth muscle actin antibodies revealed that SeV was successfully introduced into the pulmonary arterial smooth muscle cells and remained there for at least 2 weeks after the injection (Figure 2c). We measured cGMP concentrations in the treated (left) and untreated (right) lung and found that cGMP concentrations were significantly elevated in the ca*NPR2*-transduced left lungs (Figure 2d). Since whole lung lysates contain not only pulmonary vascular cells but also large amounts of other cells, the fold change observed in cGMP may have been less than that in the *in vitro* study. In order to evaluate the off-target integration of SeV vectors, we performed immunohistochemical analyses using an anti-SeV antibody. We did not detect any SeV antigens in the other organs tested including the heart, liver, spleen, kidney, and contralateral (right) lung (Figure 3). This result indicates the high specificity and safety of SeV vector–mediated intrapulmonary artery gene therapy. Taken together, these results indicate that it is possible to effectively and safely transduce SeV vectors into pulmonary arterial smooth muscles via direct injection to the intrapulmonary arteries.

### *In vivo* gene therapy with ca*NPR2* ameliorates PAH by suppressing the proliferation of vascular smooth muscle cells

**Figure 4:**
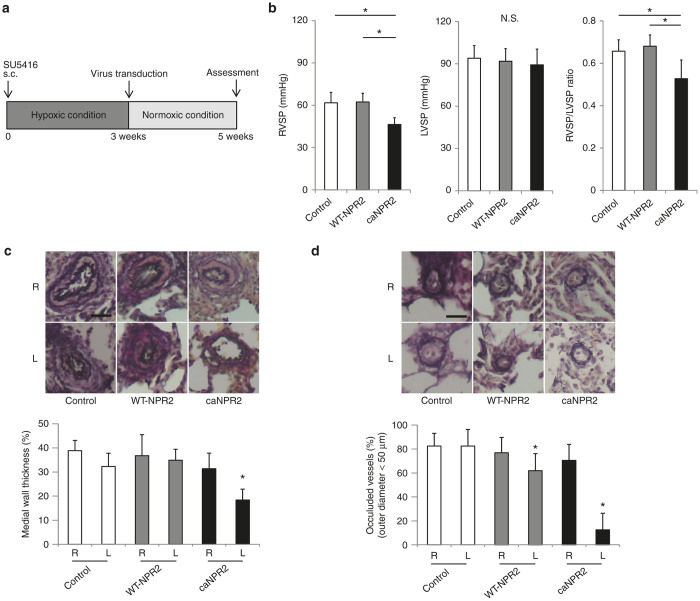
Sendai virus (SeV) vector–mediated induction of constitutively active mutant of natriuretic peptide receptor 2 (caNPR2) into pulmonary arteries hemodynamically and histologically ameliorates experimental pulmonary arterial hypertension (PAH). (a) A schematic flow chart of the gene therapy protocol for the PAH model rat. (b) Hemodynamic analyses show that the ca*NPR2* treatment significantly decreases right ventricular systolic pressure (RVSP), but not left ventricular systolic pressure (LVSP) (*n* = 8). *P < 0.05. (c) Elastica van Gieson (EVG) staining demonstrates that the thickened medial walls of the pulmonary arteries are significantly improved in ca*NPR2*-treated lungs (*n* = 8). The right lung (R: untreated lung) and left lung (L: treated lung) in each rat are analyzed. Upper panels show representative images of the EVG staining of resistant pulmonary arteries (50–200 µm in diameter). Bar = 50 µm. The lower panels show corresponding quantifications. *P < 0.05 versus control. (d) EVG staining demonstrates that the numbers of completely occluded small pulmonary capillaries (<50 µm in diameter) are significantly decreased in ca*NPR2*-treated lungs (*n* = 8). Upper panels show the representative images of pulmonary capillaries. Bar = 50 µm. The lower panel shows corresponding quantifications. *P < 0.05 versus control.

**Figure 5:**
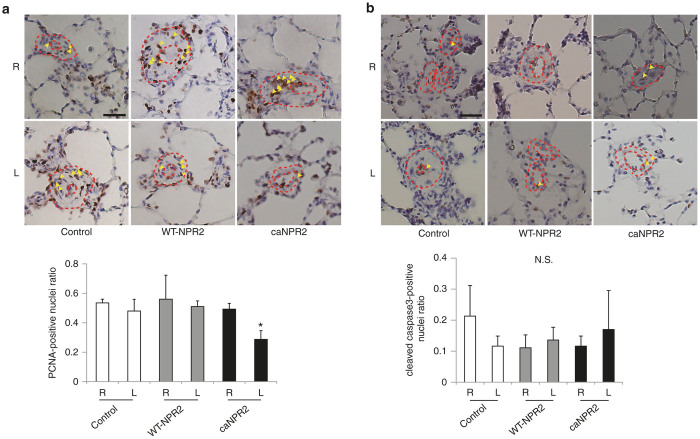
Sendai virus (SeV) vector–mediated induction of constitutively active mutant of natriuretic peptide receptor 2 (caNPR2) in pulmonary arteries attenuates the proliferation of pulmonary arterial cells in experimental pulmonary arterial hypertension (PAH). (a) Immunostaining of proliferating cell nuclear antigen (PCNA) shows that the number of proliferative cells (arrowheads) in pulmonary arterial walls is significantly decreased in ca*NPR2*-treated lungs (*n* = 4). Upper panels show representative images of pulmonary arteries, which are marked by the dashed circles. Bar = 50 µm. *P < 0.05 versus control. (b) Immunostaining of cleaved caspase-3 shows that ca*NPR2* induction does not affect cell apoptosis (*n* = 3). Positive cells are marked by arrowheads. Upper panels show representative images of the analyzed pulmonary arteries, which are marked by dashed circles. Bar = 50 µm. N.S., not significant.

In order to investigate the therapeutic effects of ca*NPR2* in PAH, we constructed a rat model using SU5416 and hypoxic exposure.^16–18^ Male SD adult rats were subcutaneously injected with SU5416 and kept in hypoxic chambers for 3 weeks. Each SeV vector was then transfected by an intrapulmonary artery injection, and rats were kept under normoxic conditions for a further 2 weeks (Figure 4a). We assessed the hemodynamic status of the rats and found that right ventricular systolic pressure was significantly decreased in ca*NPR2*-treated rats (control, 61.8 ± 7.4 mmHg; WT-*NPR2*, 62.4 ± 6.1 mmHg; ca*NPR2*, 46.4 ± 4.7 mmHg; *n* = 8; *P* < 0.01), while left ventricular systolic pressure was not affected (Figure 4b). This result suggests that the ca*NPR2* treatment effectively reduces pulmonary artery pressure, even when only the left lung is treated. Histological analyses clearly demonstrated that the medial wall thickness of the resistant artery (50–200 µm in diameter) was significantly reduced in ca*NPR2*-treated lungs (Figure 4c). The number of occluded capillary arteries was also significantly decreased (Figure 4d). In order to investigate the possible mechanism of this therapeutic effect, the proliferation of pulmonary artery smooth muscle cells was analyzed. Based on the number of proliferating cell nuclear antigen–positive proliferative cells, proliferation in the pulmonary vascular wall was significantly decreased in ca*NPR2*-treated lungs (Figure 5a). Previous research found that apoptotic vascular cells are present and play important roles in both medial and intimal lesions of the pulmonary arteries in the pathogenesis of a Sugen PAH rat model.^17,19,20^ Therefore, apoptosis was analyzed by the number of cleaved caspase-3-positive apoptotic cells. We found that the number of apoptotic cells in pulmonary vascular walls was not affected (Figure 5b).

These results are consistent with the *in vitro* results and indicate that the regression of pulmonary hypertension is attributed to the cGMP-induced attenuation of proliferation, not to the induction of apoptosis in pulmonary smooth muscle cells. Taken together, these results show that the induction of ca*NPR2* in pulmonary arteries *in vivo* ameliorates experimental PAH by inhibiting the proliferation of pulmonary arterial smooth muscle cells.

### ca*NPR2* induction effectively suppresses the proliferation of pulmonary hypertension patient-derived pulmonary arterial smooth muscle cells (PHSMCs)

**Figure 6:**
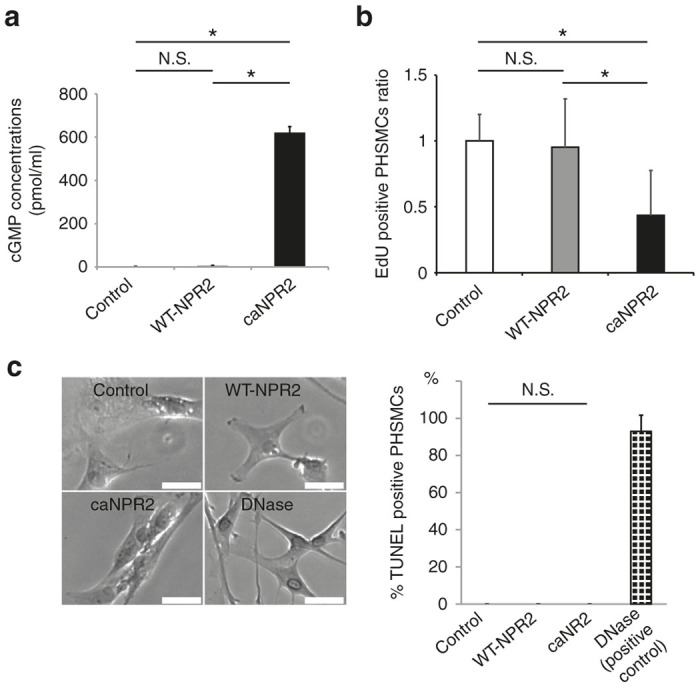
Sendai virus (SeV) vector–mediated transduction of constitutively active mutant of natriuretic peptide receptor 2 (caNPR2) suppresses the proliferation of pulmonary arterial hypertension (PAH) patient-derived pulmonary artery smooth muscle cells (PHSMCs). (a) The infection of SeV vectors carrying ca*NPR2* strongly induces the production of cyclic guanosine monophosphate (cGMP) in PHSMCs without the C-type natriuretic peptide (CNP) stimulation (*n* = 3). *P < 0.05. (b) The 5-ethynyl-2′-deoxyuridine (EdU) incorporation assay reveals that ca*NPR2* suppresses the proliferation of PHSMCs, consistent with healthy PASMCs (*n* = 6). *P < 0.05. (c) ca*NPR2* does not induce apoptosis in PHSMCs. The left panels show representative images of the TUNEL assay (*n* = 3). Bar = 500 µm. The right panel shows corresponding quantifications. N.S., not significant.

To explore the therapeutic effects of ca*NPR2* in human pathological cells, PHSMCs were isolated from an idiopathic PAH patient. We measured cGMP concentrations in PHSMCs transfected with each vector and found that ca*NPR2* more strongly induced the synthesis of cGMP than the control (control, 3.21 ± 0.378 pmol/ml; WT-*NPR2*, 6.80 ± 0.438 pmol/ml; ca*NPR2*, 621 ± 28.1 pmol/ml; *n* = 3; *P* < 0.01), which was consistent with healthy PASMCs (Figure 6a). EdU assays demonstrated that ca*NPR2* suppressed cell proliferation (Figure 6b), while TUNEL assays and Annexin-V staining showed that ca*NPR2* did not induce apoptosis (Figure 6c, Supplementary Figure S1). These results indicate that SeV-mediated ca*NPR2* transduction could be effective in the pulmonary arteries of idiopathic PAH patients and Sugen/hypoxia model rats.

## Discussion

The cGMP pathway is one of the major signaling cascades for the treatment of PAH. cGMP has the ability to not only induce simple vasodilatation but also inhibit the proliferation of and induce apoptosis in pathological pulmonary smooth muscle cells.^4,5^ Two drugs classes are available to therapeutically activate this pathway: PDE5 inhibitors and a sGC stimulator. These drugs have been shown to decrease pulmonary vascular resistance and increase exercise capacity for preferable outcomes in PAH patients.^21,22^ We recently found a novel caNPR2 when we analyzed a family case of overgrowth and bone anomalies.^11^ Since this caNPR2 mutant may more strongly enhance the synthesis of cGMP than PDE5 inhibitors and a sGC stimulator, we investigated the therapeutic effects and safety of gene therapy using ca*NPR2* in this study. 

Gene therapies for PAH have been described previously.^23^ Intravenous,^24^ intrapulmonary arterial,^25,26^ and intratracheal^27,28^ administration routes have been used as delivery methods, while adenovirus,^24,27,28^ adeno-associated virus,^25^ and HVJ envelope vectors^26^ have been reported as virus vectors.^23^ In the present study, we selected an intrapulmonary artery injection of SeV vectors for several reasons. SeV vectors are known for their high transduction efficiency. A previous study demonstrated that only a 2-minute interaction between viruses and cells was sufficient for the efficient transduction of genes into vascular tissues,^15^ which was markedly shorter than that for adenovirus vectors (~45 minutes)^29^ or adeno-associated virus vectors (similar to adenovirus vectors).^30^ Furthermore, SeV vectors are well known for their low toxicity. The findings of a phase 1/2a clinical study on SeV vectors in human peripheral arterial disease were recently reported^14^ and showed the safety of SeV vector–mediated gene therapy in the human clinical field. In addition, the selection of target cells is critical in gene therapies using cell membrane-associated receptors, such as NPR2, unlike other secreted proteins.^23^ Although previous studies demonstrated that the intratracheal administration of virus vectors successfully transduced transgenes into pulmonary smooth muscle cells, they mainly infected small vascular capillaries.^31^ Since our goal is to transduce ca*NPR2* not only into pulmonary capillaries but also resistant arteries (50–200 µm in diameter), we selected a direct intrapulmonary artery injection with clamping of the proximal artery to achieve maximal therapeutic effects. We consider this method to be easily applicable in the clinical field because a transcatheter injection with the blockade of blood flow using the inflating balloon of the Swan-Ganz catheter is a very easy and familiar technique for cardiologists. Moreover, this catheter-based transduction method is safe and minimally invasive for patients.

We evaluated the *in vitro* biological effects of ca*NPR2* induction in PASMCs. A previous study suggested that PDE5 inhibitors and a sGC stimulator increased intracellular cGMP concentrations several fold.^4,5^ In the present study, the synthesis of cGMP was 10,000-fold stronger in ca*NPR2*-expressing PASMCs than in the controls regardless of the CNP stimulation. This result was consistent with our previous findings obtained using a human kidney cell line.^11^ The potent induction of cGMP synthesis by ca*NPR2* in PASMCs and PHSMCs suppressed cell proliferation,^4,5^ but had no effect on apoptosis. This may have been because our culture conditions were not suitable for the detection of apoptosis. More importantly, we did not detect any cell death during the cultivation of PASMCs and PHSMCs despite the extremely high intracellular concentration of cGMP, which appears to support the safety of clinical gene therapy with ca*NPR2*.

We investigated the therapeutic effects of ca*NPR2* in an experimental PAH model. Recent studies reported that the combined treatment of SU5416 and hypoxia may induce a more appropriate PAH model rather than the simple hypoxia model.^16–18^ Our PAH rats exhibited moderately thickened pulmonary medial walls and the occlusion of pulmonary arterioles with neointimal formation, which are very similar characteristics to the histology of human patients (Figure 4c,d). Proliferating smooth muscle cells have recently been suggested to contribute to the development of these pathological alterations.^32^ We confirmed the successful transduction of SeV vectors into pulmonary arterial smooth muscle cells by direct infusion into pulmonary arteries without significant inflammation (Figure 2b,c). A previous study that investigated the safety of intravenous SeV injection in mice did not find evidence of inflammation in the lung histologically or changes in body weight.^33^ In a clinical trial of patients pretreated with methylprednisolone, SeV injection did not induce a significant elevation in proinflammatory cytokines.^14^ Moreover, we demonstrated that there was no obvious off-target integration of SeV (Figure 3). These results suggest that SeV-mediated gene transduction is safely and effectively accomplished by a direct injection into pulmonary arteries via a Swan-Ganz catheter in human settings. Importantly, this SeV-mediated ca*NPR2* therapy markedly ameliorated pulmonary hypertension, as revealed by hemodynamic and histological analyses. Right ventricular systolic pressure was significantly decreased by the ca*NPR2* treatment but was still higher than that of normal healthy pulmonary pressure. This result was attributed to only the left pulmonary arteries in our treatment model being cured. It is important to note that left ventricular systolic pressure was not affected, indicating the high pulmonary specificity of this ca*NPR2* induction therapy. Histological analyses clearly demonstrated that the medial wall thickness and occlusion of small pulmonary arteries were significantly improved in ca*NPR2*-treated lungs. These therapeutic effects may mainly be caused by the suppressed proliferation of, and not by the induction of apoptosis in pulmonary smooth muscle cells (Figure 5a,b). However, the induction of apoptosis may have occurred earlier than the timing of our assessment. We also detected slight improvements in pulmonary vessel occlusion in WT-*NPR2*-treated left lungs. This may have been because the overexpression of WT-*NPR2* increased the intracellular synthesis of cGMP induced by the endogenous CNP stimulation. Taken together, these results indicate that *in vivo* gene therapy using SeV carrying the ca*NPR2* mutant is safe and effective for PAH.

In our *in vitro* experiments, we found that the ca*NPR2* induced the synthesis of cGMP more strongly than a PDE5 inhibitor and a sGC stimulator in PASMCs. In addition, the proliferative ability of PASMCs with ca*NPR2* transduction was significantly suppressed (Figure 1f), while a PDE5 inhibitor and a sGC stimulator did not have significant effects, only showing a small tendency toward attenuation of cell proliferation (Figure 1g). Although future *in vivo* study is required for a comparison of the effectiveness between existing medications and our gene therapy, there is a possibility that the gene therapy using ca*NPR2* might have a greater therapeutic effect.

There are still some safety concerns associated with gene therapies for the human lungs, particularly with virus vectors. Since various effective drugs are available for the treatment of PAH patients in the current era, we speculate that our gene therapy is suitable for the most severe cases of PAH, particularly for patients who are resistant to multiple drug therapies and awaiting lung transplantation to halt or at least slow disease progression. Our immunohistochemical analyses showed that there was no detectable SeV antigen in the other organs tested. Therefore, this novel strategy may be very beneficial as a bridge therapy to lung transplantation. In such cases, the infected lung may be removed at transplantation, making it less necessary to consider the long-time sequela of virus vector transduction into the lungs. It is necessary to be cautious before proceeding to human studies because rodent disease models do not often provide a translational basis for human therapeutics. However, because the same SeV vector backbone has been evaluated for safety in human clinical trials,^14^ future clinical trials may be possible by carefully considering patient selection.

### Conclusions

The direct pulmonary artery administration of SeV vectors carrying ca*NPR2* ameliorate PAH by attenuating the proliferation of PASMCs with the intracellular synthesis of large amounts of cGMP. This novel strategy of gene therapy may be beneficial for severe PAH patients.

## Materials and Methods

All experimental protocols including *in vitro* and *in vivo* studies were approved by the Ethics Committee and Animal Experimental Faculty of Osaka University Graduate School of Medicine.

### Virus vector construction

The cDNA of HA-tagged human *NPR2* was kindly gifted by Yoshihiro Ogawa (Tokyo Medical and Dental University, Japan).^34^ The mutagenesis of human *NPR2* (2647G>A; Val883Met) was performed as described previously.^11^ A SeV vector was constructed by introducing WT-*NPR2* or ca*NPR2* cDNAs into plasmids via a ligation reaction and purchased from Medical & Biological Laboratories (MBL) (Nagoya, Japan.) A control SeV vector carrying Azami-Green was also purchased from MBL. 

### PAH model rats

We used the Sugen/hypoxia rat as a PAH model.^16–18^ Adult male Sprague-Dawley rats (Japan SLC, Shizuoka, Japan) weighing 230–270 g were subcutaneously injected with SU5416 (20 µg/g) and housed for 3 weeks in a hypobaric hypoxic chamber, depressurized to 50 kPa. They were then randomized into the following 3 groups (*n* = 8/group): control (Azami-green-expressing SeV vector-transduced); WT-*NPR2*-expressing SeV vector-transduced; ca*NPR2*-expressing SeV vector-transduced. These rats were kept in normoxic (room air) chambers for an additional 2 weeks until the day of the assessment.

### *In vivo* gene transduction

*In vivo* gene transfer was performed as described previously with some modifications.^25,26^ Briefly, rats were anesthetized with isoflurane then mechanically ventilated. Rats also received the analgesic carprofen (Rimadyl 5 µg/g, subcutaneous injection). SeV vector-containing solutions (3.5 × 10^7^ cell infectious unit/dose) carrying *Azami-Green* control, *WT-NPR2*, or *caNPR2* were injected directly into the left pulmonary artery through left thoracotomy at the fourth intercostal space with clamping of the proximal site of the injection using a Microvascular clamp (World Precision Instruments, Inc., Sarasota, FL). The clamp was released 3 minutes after the injection. The chest was closed, and rats were allowed to recover from anesthesia. There was no postoperative death.

### Hemodynamic analyses

Hemodynamic measurements were performed as described previously.^26,28^ Rats were anesthetized with isoflurane and mechanically ventilated. After small median sternotomy, right ventricular systolic pressure and left ventricular systolic pressure were measured with a 22-gauge needle and pressure transducer (MLT0699; ADInstruments, Colorado Springs, CO).

### Tissue preparation

Tissue preparation was performed as described previously with some modifications.^20^ Briefly, after the hemodynamic experiments, rats were sacrificed and the left and right lungs were excised. Both lungs were washed with phosphate-buffered saline (PBS) through a PA cannula and were distended and fixed by perfusion through a tracheal cannulation with 4% paraformaldehyde/PBS. The lungs were clamped and fixed by immersion in 4% paraformaldehyde/PBS overnight. They were then blocked and embedded in paraffin. All sections were cut at a thickness of 5 µm.^18^

### Histological analyses

Morphometric analyses of medial wall thickness and assessments of occlusive small pulmonary vessels were performed as described previously.^20^ Briefly, the percent medial wall thickness was calculated in 10 muscular arteries of resistant arteries (outer diameter of 50–200 µm) per lung with the following formula: 100 × (external diameter − internal diameter)/external diameter, by using Elastica Van Gieson staining. The occlusion score^16^ was used to assess the obstruction of small pulmonary capillaries (diameter smaller than 50 µm). We evaluated 10 arteries per lung.

### Isolation and culture of human pulmonary arterial smooth muscle cells

Human pulmonary arterial smooth muscle cells were isolated from an idiopathic PAH patient (PHSMCs) as described previously.^27^ Idiopathic PAH lung tissues were obtained from the explanted lungs during lung transplantation, and signed informed consent was obtained from the patient according to the regulations of the Ethical Committee of Osaka University Hospital. A healthy human pulmonary arterial smooth muscle cell line (PASMCs) was purchased from Lonza (Walkersville, MD). PASMCs and PHSMCs were expanded and maintained in SmGM-2 (Lonza). We confirmed the characteristics and purity of the smooth muscle cells by immunocytochemical analyses for α-smooth muscle actin and found that most were α-smooth muscle actin–positive smooth muscle cells.

### Proliferation and apoptosis assay

In the *in vitro* assay, PASMCs and PHSMCs were infected with each SeV vector at multiplicity of infection of 5 and 9, respectively, for 24 hours, and proliferation and apoptosis were assessed 48 hours after infection with or without drugs (sildenafil: 5 µmol/l; riociguat: 100 µmol/l). In order to evaluate the proliferation rate, cells were incubated with 10 µmol/l 5-ethynyl-2′-deoxyuridine (EdU) for 6 hours. Incorporated EdU was detected by an anti-EdU antibody conjugated with Alexa Fluor 488 using the EdU detection kit (Thermo Fisher Scientific, Waltham, MA). SeV-infected cells were detected by an anti-SeV antibody (MBL) with a secondary antibody of Alexa Fluor 647. The numbers of EdU-positive cells in SeV-transduced cells were automatically counted by an IN Cell Analyzer 6000 (GE Healthcare, Little Chalfont, UK). TUNEL staining was conducted in order to assess apoptosis in SeV-induced PASMCs and PHSMCs using the Takara apoptosis detection kit (Takara, Tokyo, Japan) according to the manufacturer’s instructions.

Deoxyribonuclease I-treated cells were used as positive controls. TUNEL-positive cells were manually counted, and the positive ratio was calculated.

Apoptotic cells were also evaluated by APC Annexin-V (Biolegend, San Diego, CA), according to the manufacturer’s instructions with some modifications. Camptothecin-treated cells were used as positive controls. Annexin-V-positive cells were counted by FACS Canto II flow cytometer (Becton Dickinson, San Jose, CA).

*In vivo* proliferation and apoptosis assays were performed as described previously with some modifications.^35,36^ Proliferating cells were evaluated by proliferating cell nuclear antigen staining (Santa Cruz Biotechnology, Santa Cruz, CA). Apoptotic cells were stained using a cleaved caspase-3 antibody (Cell Signaling Technology, Inc., Danvers, MA). Positive cells were visualized by 3,3′-diaminobenzidine tetrahydrochloride. The numbers of proliferating cell nuclear antigen- and cleaved caspase-3-positive cells were manually counted and evaluated as ratios to the total cell number in the pulmonary artery walls in 10 fields for each section.

### Immunocytochemistry

Cells were fixed with 4% paraformaldehyde/PBS and permeabilized with 0.2% Tween 20/PBS for 15 minutes. Cells were then incubated with 5% fetal bovine serum/PBS for 30 minutes for blocking. In order to detect SeV-transduced cells, cells were incubated at 4 °C for 16 hours with the primary antibody (anti-SeV, 1:500). Cells were then incubated for 60 minutes with the secondary antibody (Alexa Fluor 647). Nuclei were counterstained with Hoechst (Dojindo, Kumamoto, Japan). All images were taken by a LSM510 laser confocal microscope (Leica Microsystems, Wetzlar, Germany).

### Quantitative real-time PCR analyses

Total RNAs were extracted from PASMCs using NucleoSpin RNA II (Macherey-Nagel, Oensingen, Switzerland). Reverse transcription was performed using a ReverTra Ace and qPCR RT Kit (TOYOBO, Osaka, Japan). qPCR was conducted using the THUNDERBIRD Probe qPCR Mix (TOYOBO). The primer sequences used were as follows: human *GAPDH*^37^: forward primer, 5′-TGTTGCCATCAATGACCCCTT-3′, reverse primer, 5′-CTCCACGACGTACTCAGCG-3′; human *NPR2*^11^: forward primer, 5′-TTTCCGGCCAAGCATT-3′, reverse primer, 5′-GAGGTTGTCCAATATGCTGGT-3′. Gene expression levels were normalized by relative expression to *GAPDH*.

### Measurement of intracellular cGMP concentrations

Intracellular cGMP concentrations were measured in cells and rat lung tissues as described previously with some modifications.^11^ In the *in vitro* assay using SeV, SeV-infected cells were serum-starved for 6 hours or not starved on the day of the cGMP assay and were then incubated for 10 minutes with 1 mmol/l 3-isobutyl-1-methylxanthine (Wako, Tokyo, Japan) in order to inhibit the degradation of cGMP by PDE. Cells were subsequently incubated with 1 × 10^−6^ M CNP-22 (Bachem AG, Bubendorf, Switzerland) or vehicle (water) for another 10 minutes. The reaction was terminated with 100 µl of 0.1 mol/l HCl.

In the *in vitro* assay using sildenafil (5 µmol/l) or riociguat (100 µmol/l), on the day of cGMP assay (24 hours after drug stimulation), drug-treated cells were incubated for 20 minutes with 1 mmol/l 3-isobutyl-1-methylxanthine in order to inhibit the degradation of cGMP by PDE. The reaction was terminated with 100 µl of 0.1 mol/l HCl. 

In the *in vivo* assay, fresh rat lungs were placed in 5% trichloroacetic acid (Wako), fractured by MicroSmash MS-100 (TOMY, Tokyo, Japan), and then centrifuged at 15,000 rpm for 10 minutes.

All cGMP concentrations were measured using the competitive enzyme immunoassay kit (Cayman Chemical, Ann Arbor, MI) according to the manufacturer’s instructions. cGMP concentrations in lung tissues were normalized by the protein concentrations of the samples.

### Western blotting

SeV-infected cells were lysed with modified radioimmunoprecipitation buffer containing a protease inhibitor mixture (Roche Diagnostics, Basel, Switzerland). Equal amounts of protein (10 µg) were subjected to electrophoresis using 10% sodium dodecyl sulfate–polyacrylamide gels. Proteins were transferred to polyvinylidene difluoride membranes, washed with Tris-buffered saline containing 0.05% Triton X-100, and incubated with BlockingOne solution (Nacalai Tesque, Kyoto, Japan) for 60 minutes. Anti-HA tag (1:500; Cell Signaling Technology) or anti-SeV (1:2,000; MBL) was used as the primary antibody. Horseradish peroxidase-conjugated anti-mouse or anti-rabbit IgG antibodies (Promega, Madison, WI) were used as the secondary antibody. Signal Booster (Beacle, Kyoto, Japan) was used for diluting anti-HA tag antibody and the secondary antibody. As a control, β-actin was detected with an anti-β-Actin pAb-HRP-DirecT (1:2,000; MBL). Blots were visualized using Chemi-Lumi One L (Nacalai Tesque).

### Statistical analyses

All statistical analyses were performed with R version 2.14.0 (http://www.r-project.org) software. Comparisons of the results obtained from cGMP measurements in control cells and drug-treated cells (riociguat or sildenafil) were made by the two-tailed Student’s *t*-test. Multiple comparisons were evaluated by a one-way ANOVA with Tukey’s honest significant difference test. *P* < 0.05 was considered to be significant. All data and graph bars are expressed as the mean ± standard deviation.
