# Supplementary figures and images for: Constitutively active form of natriuretic peptide receptor 2 ameliorates experimental pulmonary arterial hypertension
Source: Mol Ther Methods Clin Dev. 2016 Jul 6;3:16044–. doi: 10.1038/mtm.2016.44 (PMC4934588; doi:10.1038/mtm.2016.44)

Supplementary Figure S1

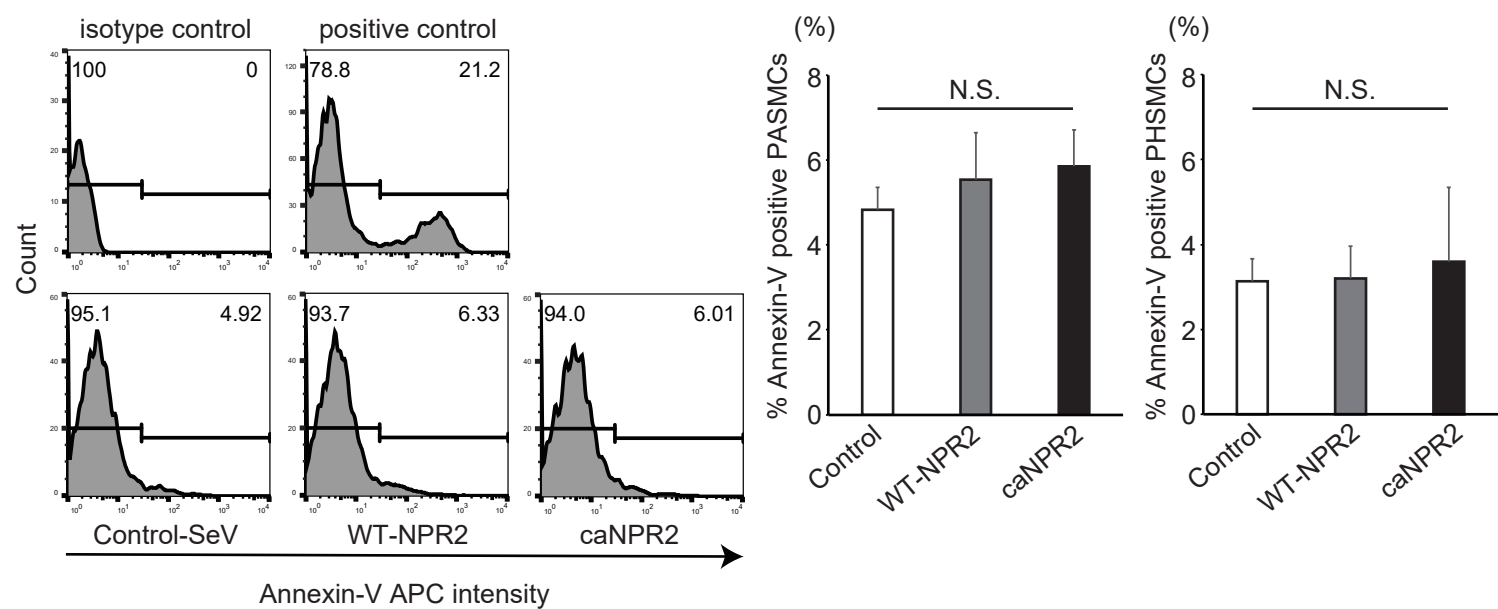

Supplement: Supplementary Figure [file mtm201644-s1.pdf]
